# Teachers' ideas versus experts' descriptions of 'the good teacher' in postgraduate medical education: implications for implementation. A qualitative study

**DOI:** 10.1186/1472-6920-11-42

**Published:** 2011-06-28

**Authors:** Thea CM van Roermund, Fred Tromp, Albert JJA Scherpbier, Ben JAM Bottema, Herman J Bueving

**Affiliations:** 1 Department Primary and Community Care, Radboud University Nijmegen Medical Centre, Nijmegen, Postbus 9101, Route number 166, 6500 HB Nijmegen, the Netherlands; 2 Department Primary and Community Care Radboud University Nijmegen Medical Centre, Nijmegen, the Netherlands; 3 Institute for Medical Education, Faculty of Health, Medicine and Life Sciences, Maastricht University, Maastricht, the Netherlands; 4 Department Primary and Community Care, Radboud University Nijmegen Medical Centre, Nijmegen, the Netherlands; 5 Department of Post Graduate Medical Training for Family Medicine, ErasmusMC, University Medical Center Rotterdam, Rotterdam, the Netherlands

## Abstract

**Background:**

When innovations are introduced in medical education, teachers often have to adapt to a new concept of what being a good teacher includes. These new concepts do not necessarily match medical teachers' own, often strong beliefs about what it means to be a good teacher.

Recently, a new competency-based description of the good teacher was developed and introduced in all the Departments of Postgraduate Medical Education for Family Physicians in the Netherlands. We compared the views reflected in the new description with the views of teachers who were required to adopt the new framework.

**Methods:**

Qualitative study. We interviewed teachers in two Departments of Postgraduate Medical Education for Family Physicians in the Netherlands. The transcripts of the interviews were analysed independently by two researchers, who coded and categorised relevant fragments until consensus was reached on six themes. We investigated to what extent these themes matched the new description.

**Results:**

Comparing the teachers' views with the concepts described in the new competency-based framework is like looking into two mirrors that reflect clearly dissimilar images. At least two of the themes we found are important in relation to the implementation of new educational methods: the teachers' identification and organisational culture. The latter plays an important role in the development of teachers' ideas about good teaching.

**Conclusions:**

The main finding of this study is the key role played by the teachers' feelings regarding their professional identity and by the local teaching culture in shaping teachers' views and expectations regarding their work. This suggests that in implementing a new teaching framework and in faculty development programmes, careful attention should be paid to teachers' existing identification model and the culture that fostered it.

## Background

In times of change in medical education, teachers are often asked to adapt to a new concept of what being a good teacher includes [1,2]. These new concepts represent what is considered to be best educational practice and are often developed by experts outside the departments where the changes are to be implemented. The concepts are based on an analysis of the teachers' tasks and/or educational theory and operationalized in competency profiles and evaluation questionnaires [3-11]. However, teachers have their own ideas about what it means to be a good teacher, and these ideas are not necessarily in line with new concepts [2,12,16,20]. When new concepts and teachers' views are too widely divergent, teachers may feel unable to fulfil their new role. This can cause feelings of guilt leading to resistance and loss of self-confidence and motivation [2,16,20]. This is clearly a most undesirable effect that should be considered by managers who want to implement new educational methods and trainers who design faculty development programs in order to achieve the new competencies[12,24-28].

Whereas experts' ideas are mainly theory based, teachers' ideas about good teaching tend to be mainly rooted in their own educational experiences and memories, which are context based and generally rather unstructured [20,29]. Subsequently, teachers' ideas are strongly influenced by feedback from other stakeholders in educational practice: colleagues, administrators and trainees. Some ideas are confirmed, others are criticized. Teachers reflect upon feedback, asking themselves: "is it true what others are saying about me" [20,30-32]. They also think about the effectiveness of their teaching in terms of their trainees' learning outcomes [29,33,34]. Gradually, teachers' ideas become strong convictions to which they cling to in times of change.

This study addresses the conflict between teaching concepts in a new competency profile that was introduced and teachers' views on teaching in the postgraduate programmes for family medicine in the Netherlands (Table 1). The programmes consist of two main strands: work-based learning with a family physician as supervisor and a department-based day release programme. Traditionally, the teachers of the day release programme are family physicians and psychologists. They are appointed as group coaches and combine part-time teaching with their work as a practising family physician or psychologist. The programme consists of weekly small group sessions in which trainees reflect on scientific topics and their personal functioning. They also practise communication skills and share experiences. In the new teaching competency framework, the distinction between family physician- as- teacher and psychologist- as- teacher is only noticeable in relation to competency 0 (Role as an expert).

**Table 1:** The new competency profile of the teacher in postgraduate education for family physicians

Dimensions	Competencies and teaching behaviours
**Dimension 0: Role as an expert** **The teacher acts in accordance with the competencies defined by the profession**	

0.0	The teacher is a role model for the various competencies of a family physician
	- Serves as a role model for professional competencies. - Articulates and explains the motives for his/her actions.

0.1	Teachers are role models in curriculum units related to their particular specialty (family physician or behavioural scientist)
	The teacher models the behaviour and competencies of a professional practitioner - Articulates and explains the motives for his/her actions - Models the behaviour and competencies of his/her specialty as a behavioural scientist - Explains the actions of a specialist.

**Dimension 1: Role as an educator of adults** **The teacher achieves and maintains a constructive learning climate to help trainees develop their professional competencies.**	

1.1	The teacher creates and maintains a constructive learning climate in which trainees can develop into self-directed and competent professionals.
	- Stimulates respectful interpersonal behaviour. - Stimulates trainees to make use of one another's experiences and competencies. - Monitors the interaction between individual learning processes and group processes.

1.2	The teacher builds a constructive relationship with individual trainees.
	- Creates a safe environment in which trainees are stimulated to discuss intellectually and emotionally challenging situations. - Challenges trainees to engage in dialogue.

1.3	The teacher is able to deal with diversity in an appropriate way.
	- Is sensitive to trainees' cultural, social and ethnic backgrounds. - Uses differences between trainees as learning opportunities.

**Dimension 2: Role as a teacher** **The teacher creates and maintains a powerful learning environment**	

2.1	Provides a balanced and challenging programme.
	- Develops a series of learning activities in alignment with both the curriculum and professional practice. - Stimulates learning processes and applies appropriate educational methods. - Shows flexibility in using different levels of guidance in supporting the learning process. - Monitors and evaluates trainees' learning processes and outcomes.

2.2	Coaches trainees to become self directed in pursuing their learning goals.
	- Supports trainees in formulating an individual learning plan. - Supports trainees in formulating learning goals and strategies to achieve them. - Identifies ineffective learning strategies and explains why they are ineffective. - Challenges trainees to move outside their comfort zone and discusses the feasibility of trainees' plans. - Stimulates and monitors trainees in taking responsibility for their own learning.

2.3	Evaluates results and discusses the consequences in relation to trainees' progress.
	- Evaluates the quality of the portfolio. - Provides objective and concrete feedback on learning results. - Evaluates and documents the progress of trainees' learning. - Advises the head of the department about trainees' progress. - Takes measures to ensure the continuity of trainees' learning trajectories.

2.4	Develops materials for education and assessment appropriate for a competency-based curriculum.
	- Designs programmes, assignments and assessments in accordance with current educational principles. - Writes texts that are fit for purpose and tailored to the target group. - Ensures that documents are uniform with regard to structure and lay-out. - Discusses development processes with all stakeholders. - Adjusts programmes, assignments and assessments based on the results of systematic evaluation.

**Dimension 3: Collaboration** **The teacher promotes the realization and optimization of the training programme by consulting with all those involved.**	

3.1	The teacher uses collaborative skills in a purposeful way...
	- Collaborates with other teachers and colleagues to ensure clarity and agreement with regard to content and execution of tasks and responsibilities. - Makes a constructive contribution as a member of groups with different goals and memberships. - Identifies problems and supports trainees in learning how to solve (threatening) conflicts. - Takes responsibility for the consistency and continuity of the education programme.

3.2	The teacher coordinates his/her actions with all those involved in supporting trainees.
	- Takes responsibility for the work done by the triangle teacher-trainee-supervisor. - Is able to stimulate other supervisors and teachers to contribute effectively to the education of trainees. - Provides information about trainees' performance effectively and confidentially.

**Dimension 4: Organization** **The teacher's actions are directed at effective management of time, information, means and people.**	

4.1	The teacher organizes information transmission, means and materials to ensure efficient delivery of the education programme.
	- Ensures timely availability of materials and equipment for education. - Ensures that up to date information is provided in an effective way.

4.2	The teacher acts in accordance with the relevant regulations and guidelines of the department.
	- Acts in accordance with the relevant regulations designed by the board of governors. - Acts in accordance with the legal framework for training programmes for family physicians - Acts in accordance with the guidelines of the department.

**Dimension 5: Professionalism** **The teacher maintains and develops his/her competencies as a teacher.**	

5.1	The teacher demonstrates his/her personal involvement in education.
	- The teacher's behaviour inspires trainees to learn. - Clearly enjoys working with trainees. - Acts as an ambassador of the department.

5.2	The teacher engages in improving his/her professional performance in a systematic and purposeful way.
	- Is prepared to discuss his or her personal and professional performance and proposes improvements based on feedback received. - Regularly identifies personal development needs based on self reflection. - Participates in discussions and in the development of new insights in relation to the professional field of education. - Uses scientific insights and 'best practices' to direct his or her work in education.

5.3	The teacher monitors the balance between involvement and distance.
	- Is able to make an adequate distinction between work-related and personal aspects in the work environment.

5.4	The teacher applies the various roles in a flexible way.
	- Uses appropriate roles in different contexts.

5.5	The teacher acts in accordance with the rules of ethical behaviour and social relations.
	- Shows integrity and is scrupulous in using his or her influence on individual trainees and groups of trainees.

Based on anticipated resistance to the implementation of the new competency profile and looking for ideas for an effective faculty development programme, we sought the coaches' views on the good teacher and compared these with the views reflected in the new competency framework.

Our aim was to answer two research questions:

1. What are teachers' ideas of 'the good teacher'?

2. To what extent do teachers' views correspond with the concept of 'the good teacher' described in the new competency profile?

## Methods

### Design

Because of the open character of the first research question, we conducted a qualitative interview study [35], involving semi-structured individual interviews with teachers. The interviews were tape-recorded, transcribed and analysed using software for qualitative research (Kwalitan).

In order to address the second research question, we examined the match between the competencies in the framework and the themes that emerged from the interviews by linking the views of the teachers to the competencies in the framework (TvR) and discussing the links with two researchers (TvR and FT) until consensus was reached.

### Interview framework

We searched the literature for methods to explore teachers' views[13-15,17,21,22].. We used an interview framework based on grounded theory that was used in a study of teachers' professional development by Kelchtermans[14,15]. From this framework four interview topics were derived: self image, job motivation, task perception and the teachers' perspective with regard to the future of the training programme. A fifth topic, used as the opening topic of the interviews, related to teachers' initial experiences. Three pilot interviews were conducted to determine whether the framework was effective in eliciting teachers' views of good teaching. These pilot interviews were not included in the analysis. One of the authors (AS) checked the pilot interviews and judged that the information obtained was relevant. The interview framework was used for all the interviews (Table 2).

**Table 2:** Interview framework

Central topic: beliefs of teachers regarding their competencies		
**Code**	**Date and place**	**Details**
	
**Topics and sub topics (if necessary)**		

**1. Start of the teaching profession**		
- Your first day as a teacher in the department		
- Expectations and outcomes		
- Critical events and persons		

**2. Professional identity: who are you as a teacher**		
- The concept of group coach		
- Most important educational values and aims		
- What do and what do trainees not learn in your classes		

**3. Motivation to teach**		
- What makes you come here every week		
- Challenges and limitations in the department		
- What makes your day a good day when you are teaching		

**4. Views regarding important competencies**		
- Considerations about good teaching		
- What should a teacher do and not do		
- Relationship with trainees		

**5. The future and the role of the teacher**		
- The most important changes of the past years		
- Trends for the next ten years		
- Your role in the future of the training programme		

### Recruitment

Teachers from two departments were invited to take part in the study. All participants received written assurance that the interviews would be analyzed anonymously and confidentially. They all agreed to participate and gave informed consent.

We first interviewed all teachers of one department and then continued to interview teachers from the other department until saturation of information was reached. Participation was voluntary and the teachers determined the location of the interview. The interviews lasted one hour and were taken in a quiet room in the interviewee's department, except for one interview which, at the teacher's request, was conducted in the teacher's practice. All interviews were audio recorded and the recordings were erased after a transcript and an anonymous summary were made. Notes taken by the interviewers were used to determine whether new information was emerging.

### Analysis

The transcripts were entered into qualitative data analysis software (Kwalitan). Two researchers (TvR and FT) conducted the analysis. After independently coding the transcripts, they discussed the coding until full agreement was reached. Controlling for synonyms and discussion about duplicates led to a total of 161 different codes. Next, the researchers identified fragments with relevance to the first research question, explored connections between the fragments and categorized the codes. In a process of constant comparison of codes and categories, a tree structure was developed and unrelated codes were discarded, all by consensus. Finally, the researchers discussed their analyses and agreed on the final six themes. After the themes were summarized, the co researcher (FT) and the co-authors (AS, BB and HB) gave feedback on the fragments which the first author proposed to use in determining a match with the competency profile and to illustrate the results. For each theme, two or three fragments were selected by consensus.

### Comparison

We addressed the second research question by matching the teachers' views of the 'good teacher' that emerged from the interviews with the teaching competencies described in the competency profile (Table 3). First, the views of the teachers were summarized for each theme. We then identified which competencies of the competency profile matched the teachers' views and summarized these.

**Table 3:** Example

Theme: Relationship with trainees	Teachers' views	**The competency profile (Table **1**)**
Data from interviews and competency framework	As a group coach you need to hear what they really need. You feel responsible for their well being. Alternate between the father and the mother role.	1.1 Helping trainees develop self direction and competency. 5.3 Balancing between involvement and distance. 5.4 Applying various roles in a flexible way 1.1 Monitors the interaction between individual learning processes and group processes 1.2 The teacher builds a constructive relationship with individual trainees

Summary	Emphasis on caring roles. Building a close relationship. Position of coach in the group.	Alternating between various forms of regulation, monitoring individual and group learning processes, professional distance to trainees

## Results

### Participants

Table 4 shows the characteristics of the teachers who participated in the interviews.

**Table 4:** Characteristics of the teachers who participated in the interviews

Characteristics	Description
Men	18

Women	10

Family medicine - teacher	18 (3 women/15 men)

Behavioural Sciences - teacher	10 (6 women/ 4 men)

Age	30-60 years

Experience as a teacher	1-20 years

### Interviews

The information from the answers of the teachers could not be adequately captured by the interview topics as they yielded more, and more varied information than expected. The analysis resulted in six themes that reflected the data: professional identification, relationship with trainees, learning through socialization, knowledge, motivation to teach and change.

We present the results for each theme with illustrative quotes from family physicians-as-coaches (FP) or psychologists-as-coaches (P).

### Research question 1: What are the teachers' ideas of 'the good teacher'?

#### Theme 1 Professional identification

The teachers indicated that the word 'teacher' evoked ideas of authority, unequal relationships and the role of an expert, which they felt were not consistent with how they saw their role as a group coach. They were more comfortable with an interpretation of their role as that of a wiser colleague who helps trainees in reflecting on their experiences.

Teachers expressed doubts as to whether they were able to teach trainees anything new. When they first took up the coaching role, they did not always have a clear view of what it involved.

- *'Teacher' evokes an image of standing in front of the. I don't really feel that I have anything to offer that trainees haven't already experienced. I have always worked from a group coach perspective, helping trainees reflect on their work. I still can't say the word: 'teacher' (P22).*

- *I have to get used to this academic position. I didn't realize this when I first took this job as a group coach. (FP18).*

#### Theme 2 Relationship with trainees

On the one hand the teachers wished to look upon trainees as adults and highly educated future colleagues whose expectations they wanted to meet. They felt that the best way to achieve this was by acting as a 'group coach', facilitating reflection on experiences. On the other hand, the teachers also saw themselves in a sort of parental role, based on their conviction that they knew better than the trainees what was good for them.

- *Trainees see me first and foremost as a group coach. But when I turn out to be an expert on some subject, then they suddenly see me as a teacher (FP17).*

- *With my co-teacher, as a team, we alternate between the father and mother role. You feel responsible for their well being (FP5).*

- *When trainees say 'we know what we need as future family physicians', you must hear what they really need. (P11)*

#### Theme 3 Learning to be a teacher

Teachers in departments of family medicine learn from and with each other. Every new teacher is initially mentored by an experienced colleague. All the interviewees had learned how to be a teacher within the specific culture of their department and could name some colleagues that had been important for their development or had a dominant position in the department.

- *We hardly ever consult experts, so, the level of teaching never exceeds that of the best teacher in the group (P24).*

- *My mentor was very dominant. I adopted the way we do things around here. (P7).*

- *I learned a lot from X. He always came up with something new to discuss with the trainees. Just like that (snapping his fingers) (FP8).*

#### Theme 4 Knowledge

The teachers viewed themselves as coaches rather than medical experts. They did not think they were expected to show medical knowledge, but emphasized the importance of their knowledge about group dynamics. Expertise in doctor patient communication was also an area they considered important for teaching, as well as enthusiasm for the profession and building relationships with patients.

- *As their teacher I don't have to know all the ins and outs of medicine, such as how to treat all kinds of cardiovascular problems in family medicine practice (FP26).*

- *Well, the main thing is communication with patients. I think that's one of my specialties (P27).*

- *You have to know about group dynamics. One group was very difficult to handle and then I began to doubt my abilities as a group coach. Fortunately, the chemistry with the next group was good (FP3).*

#### Theme 5 Motivation to teach

The main reasons the teachers gave for being a postgraduate teacher were: witnessing the trainees' personal development, feeling a member of a guild and a welcome diversion in a busy working week in their own practice. The teachers felt that their work would be more interesting if they were given a certain level of autonomy in their teaching and they expected the department to give them that autonomy. Most of the teachers were inspired just by working with trainees but others looked for other challenges and teamwork.

- *I teach because it gives me satisfaction. You see trainees struggling for answers. That's real synergy! But I need to be challenged. If that's missing, I get bored (P1).*

- *The institute has to allow you to do the things you think are important (FP8).*

#### Theme 6 Change

Recently, educational changes were implemented in the departments and new teaching behaviours were introduced by the departmental management teams. The teachers experienced these changes as just more work, because they no longer had a clear view of what was expected of them. It is not only changes within the department but also changes within the teachers themselves that influence teachers' work with trainees.

- *I think there are too many changes that are enforced by departmental management. When a new project is launched, what are we supposed to stop doing? But that's never a point of discussion (P25).*

- *The problem is that I have to give out a message that is not mine. That diverts attention away from the things we should really be doing (FP16).*

- *At first I was a lecturer but now I prefer a more consultative style (FP5).*

### Research question 2: Comparison of the teachers' views and the views of the competency-based model

In order to address the second research question, we identified which competencies of the competency profile matched the teachers' views and summarized these in Table 5.

**Table 5:** Summary of Teachers' views

Themes	Teachers' views	**Competency profile (Table **1**)**
1. Professional identification	Teachers are comfortable with the concept of 'group coach' in the sense of a wiser and more experienced colleague.	The concept of teacher is consistently used with reference to all persons involved with trainees. (1.1 t/m 5.5)

2. Relationship with trainees	Emphasis on caring roles, Takes the position of coach in the group. Builds up a close relationship with individual trainees.	Alternating between various forms of regulation, and professional distance to trainees. (1.1;5.3;5.4) Monitors the interaction between individual learning processes and group processes (1.1) The teacher builds a constructive relationship with individual trainees (1.2)

3. Learning to be a teacher	Learning to be a teacher is mostly achieved by learning by doing and from your fellow group coach and mentor.	Based on feedback and systematic reflection on development needs. Oriented to scientific insights, using 'best medical education practices' (5.2)

4. Knowledge base	Practical experience and knowledge about group dynamics are the basics for a successful course.	Knowledge is assimilated in the description of competencies. As a role model, the teacher acts in accordance with the competencies defined by the profession

5. Motivation	Seeing trainees grow as professionals and being away from a busy practice.	Clearly enjoys working with trainees. (5.2)

6. Change	Changes are often imposed by others than the teachers and obscure the teacher's vision of what they should and should not do..	Participates in discussions and in the development of new insights in relation to the professional field of education (5.2)

## Discussion

We investigated how postgraduate teachers, who develop and facilitate small group sessions for postgraduate trainees in family medicine practice, view 'the good teacher' and compared these views with the description of 'the good teacher' in a recently introduced competency-based teaching framework.

The teachers that took part in this study are family physicians and behavioural scientists who act as a team in coaching small groups of family physician trainees. They were interviewed as one group of teachers because the competency framework refers to a general concept of teachers and distinguishes family physicians and psychologists only in the expert dimension. Though their backgrounds differ, we did not find differences in their beliefs about teaching. The family physicians and the behavioural scientists see themselves as health care providers. This seems to unify them in their beliefs about teaching and their identification with their role as coaches. Coaching is felt as closely related to their roles in practice as family physicians or psychologists.

When we compare the teachers' views with the concepts described in the competency framework, we seem to be looking in two mirrors that reflect clearly dissimilar images. When they look into a new mirror, teachers truly believe they could do better and indicate that they would like to enhance their competencies, but at the same time they hold on to the beliefs and methods they have learned through experience. In this situation, training by holding up a new competency framework does not automatically lead to acceptance of a new concept of teaching. It also does not automatically lead to the demonstration of the desired competencies. Clearly, there are other processes to be considered as well.

Two major processes appear to have the greatest relevance from the perspective of implementation of new educational methods. The first process is that of identification [36,37]. This is an interesting issue which we were not aware of when we started this study. For the teachers, the word teacher conjured up an image that held little attraction for them: an authority who teaches trainees new knowledge and skills. Coaching feels more familiar and is closely bound up with their role as a health care professional. In the literature this is known as "work-group identification" [37]. This means that professionals tend to identify with the values and demands of their profession rather than with those of a different organization, such as the department of medical education[20,36,37]. The second process that plays an important role is that of the organizational culture. As soon as a family physicians or a psychologist is appointed as a group coach, experienced mentors start up a socialization process which shapes their new professional development as a teacher. In such a learning environment, the new teacher not only learns 'how to teach', but is also initiated into the do's and don'ts of teaching in the local departmental culture. Literature confirms that the impact of educational innovations on teachers is not a purely individual process but occurs in interaction with others [2,30,31]. The loss of certainties and confrontation with the unfamiliar are a source of concern. Trainers and leaders who influence and coordinate these very complex innovation processes should be mindful of the fact that new teaching behaviour is not only a matter of 'can or cannot' but also a matter of value and norms related choices of the teachers involved [2,20].

## Conclusions

The main findings of this study are the key roles played by feelings regarding professional identity and the local teaching culture in shaping teachers' views and expectations with regard to their work. This can be interpreted as an important message for managers implementing educational change and for designers of faculty development programmes. Before, during and after the implementation of new educational methods, careful attention should be paid to teachers' existing identification model and the culture that fostered it. Similarly, faculty development programmes should not only focus on teaching 'new' concepts but also on discussing the 'old' concepts of teaching.

In view of the importance of the departmental teaching culture that emerged from this study we recommend further studies to investigate the design and effects of teachers' learning environments analogous to studies of the learning environments of students and trainees.

## Limitations and strengths of the study

A limitation of this study is that we studied a rather small population, because qualitative research is time consuming as it requires detailed analysis of a large amount of fairly unstructured data. It is a strength of this study is that we took time to collect data until saturation was reached. We also took time to listen to teachers' opinions, which yielded unexpected and valuable insights into their views on teaching.

## Competing interests

The authors declare that they have no competing interests.

## Authors' contributions

TvR was principal researcher and wrote the article. FT was co-researcher and contributed to the data analysis. AJJAS was co-author and supervisor during the data collection and the analysis process and gave feedback on the selection of quotations. BJAMB was co-author and gave feedback on the selection of quotations. HJB was co-author and gave feedback on the selection of quotations.

All authors read and approved the final manuscript.

## Pre-publication history

The pre-publication history for this paper can be accessed here:

http://www.biomedcentral.com/1472-6920/11/42/prepub
